# Bridging the Gap Between Treatment and Accountability: DR-TB Outcomes and Governance Signals in Rural Eastern Cape, South Africa (2020–2024)

**DOI:** 10.3390/idr18050099

**Published:** 2026-09-07

**Authors:** Kamvelihle Sabisa, Lindiwe Modest Faye, Ntandazo Dlatu, Teke Ruffin Apalata

**Affiliations:** 1 Walter Sisulu Institute for Clinical Governance, Healthcare Administration, School of Public Health, Faculty of Medicine and Health Sciences, Walter Sisulu University, Mthatha 5099, South Africa; 207616620@mywsu.ac.za (K.S.); ndlatu@wsu.ac.za (N.D.); 2 TB Research Group, School of Pathology, Faculty of Medicine and Health Sciences, Walter Sisulu University, Mthatha 5099, South Africa; tapalata@wsu.ac.za

**Keywords:** tuberculosis, drug-resistant TB, treatment outcomes, not evaluated, health system governance, routine surveillance data, programme accountability, rural South Africa

## Abstract

**Background**: Monitoring treatment outcomes is central to evaluating drug-resistant tuberculosis (DR-TB) programmes and ensuring accountability, particularly in high-burden settings such as South Africa. However, routine surveillance data are often constrained by small sample sizes and incomplete reporting. The increasing proportion of outcomes classified as “not evaluated” raises critical concerns about the validity and interpretability of reported treatment success rates. This study examines longitudinal trends in DR-TB outcomes in two rural Eastern Cape facilities (2020–2024), conceptualising routine outcomes as governance signals reflecting health system performance and data management capacity, rather than clinical performance alone. **Methods**: A retrospective analysis of aggregated DR-TB treatment outcomes from 2020 to 2024 was conducted using routine programme data. Outcomes included treatment success, failure, loss to follow-up (LTFU), death, and “not evaluated.” Proportions were estimated using Wilson 95% confidence intervals, and year-to-year differences were assessed using chi-square tests, with cautious interpretation due to small sample sizes. Bounding analyses were applied to assess the sensitivity of treatment success estimates to alternative assumptions about incomplete outcome reporting. **Results**: Treatment success declined over time for both rifampicin-resistant TB (RR-TB) and multidrug-resistant TB (MDR-TB), with a significant decrease observed in MDR-TB (80.9% in 2021 to 38.5% in 2024; *p* = 0.007). Concurrently, the proportion of outcomes classified as “not evaluated” increased substantially from 2023 onward, reaching 25.8% in RR-TB and 30.8% in MDR-TB by 2024 (*p* < 0.001). Mortality remained persistently high in RR-TB, while MDR-TB exhibited higher levels of treatment failure and LTFU. Analyses for pre-XDR-TB and XDR-TB were limited by small sample sizes. Bounding analyses demonstrated that treatment success estimates were highly sensitive to incomplete reporting of outcomes. **Conclusions**: Declining DR-TB treatment success in this setting is closely associated with increasing incomplete outcome reporting, suggesting that observed trends may reflect challenges in surveillance and governance systems rather than clinical deterioration alone. Interpreting routine DR-TB outcomes as governance signals provides a novel systems-based perspective for programme evaluation. Strengthening data completeness, patient tracking, and surveillance systems is essential to improve the reliability of programme indicators and support effective TB control in resource-constrained rural settings.

## 1. Introduction

Drug-resistant tuberculosis (DR-TB), including rifampicin-resistant TB (RR-TB), multidrug-resistant TB (MDR-TB), and extensively drug-resistant TB (XDR-TB), remains a major global public health challenge despite advances in diagnostics and treatment [1,2]. Globally, treatment success rates for DR-TB remain substantially below the targets set by the World Health Organization (WHO) End TB Strategy, particularly in high-burden and resource-constrained settings [3]. South Africa continues to carry one of the highest burdens of DR-TB worldwide, with persistent gaps in treatment outcomes and programme performance [2,4]. Health system capacity to manage DR-TB is unevenly distributed across urban and rural settings in low- and middle-income countries [1,5]. Urban tertiary facilities typically benefit from greater specialist availability, stronger diagnostic infrastructure, and more robust patient tracking systems. In contrast, rural health systems are often constrained by workforce shortages, long travel distances, fragmented referral pathways, and limited surveillance capacity [6,7]. In provinces such as the Eastern Cape, these structural challenges are further compounded by socioeconomic vulnerability and health system fragmentation, undermining continuity of care and effective programme monitoring [4]. The growing prevalence of Pre-XDR-TB and XDR-TB, which are the most severe forms of drug resistance, is particularly concerning. These forms are associated with poorer treatment outcomes, including lower success rates, increased mortality, prolonged infectiousness, higher healthcare costs, and greater challenges for tuberculosis control programmes [8,9]. These burdens are disproportionately experienced in rural and resource-constrained settings, where access to specialist care, advanced diagnostics, and comprehensive patient monitoring remain limited [9,10,11]. Despite the growing burden of MDR-TB, Pre-XDR-TB and XDR-TB, evidence regarding treatment outcome monitoring and surveillance performance among patients with advanced forms of drug resistance remains limited in rural South African settings [12,13]. Monitoring DR-TB treatment outcomes is central to evaluating programme performance and ensuring accountability. Routine surveillance systems, including national platforms such as the Electronic DR-TB Register and the District Health Information System, are designed to track patient outcomes and inform programme management. However, these systems are frequently limited by incomplete reporting, delayed data entry, and inconsistencies between facility-level and aggregated data. The rising proportion of outcomes classified as “not evaluated” has become a critical concern, undermining the validity and interpretability of reported treatment success rates. At both national and global levels, incomplete outcome ascertainment limits the ability to accurately assess programme performance and progress toward TB control targets [2]. Existing research on DR-TB outcomes has largely focused on patient-level determinants, including clinical characteristics, treatment regimens, and adherence factors [1,7,10]. While these studies have provided important insights into predictors of treatment success and failure, they often assume that routine outcome data accurately reflect clinical performance. Less attention has been given to the role of surveillance systems, data completeness, and governance processes in shaping observed outcome patterns, particularly in decentralized and resource-constrained settings [2,5]. Consequently, apparent trends in treatment success, mortality, and loss to follow-up (LTFU) may be confounded by underlying weaknesses in data systems and programme accountability. In this context, DR-TB outcomes can be conceptualized not only as clinical endpoints but also as system-level outputs co-produced through interactions among patients, healthcare providers, and health system structures [1,5]. Gaps in patient tracing, follow-up, and data integration may lead to incomplete outcome ascertainment, distorting programme indicators and obscuring the true relationship between care delivery and reported outcomes. This challenge is particularly relevant in decentralized models of care, where expanded access to treatment has not always been matched by corresponding improvements in clinical governance and surveillance systems [14,15]. From a health systems and implementation science perspective, routine programme indicators are shaped not only by clinical events but also by the functioning of service delivery, information systems, accountability structures, and feedback mechanisms. In tuberculosis programmes, outcome completeness depends on whether patients are retained in care, transfers are documented, deaths are recorded, and final outcomes are assigned and reported through surveillance systems. Therefore, incomplete outcomes such as “not evaluated” may reflect more than missing data; they may indicate operational gaps in tracing, documentation, inter-facility communication, and responsibility for outcome assignment. Health systems frameworks emphasize that reliable information, accountability, continuity of care, and adaptive learning are core components of effective programme performance. Similarly, implementation science highlights that gaps between policy and practice often become visible through failures in routine processes, including incomplete documentation, delayed reporting, and weak follow-up systems. In this study, the interpretation of “not evaluated” outcomes as potential governance signals is therefore grounded in the view that surveillance data are produced through health system processes and can reveal weaknesses in programme implementation and accountability. This study is informed by a CE–CG framework, which integrates principles of clinical governance, community engagement, surveillance quality, and programme accountability [2,16]. Within this framework, DR-TB outcomes are interpreted as products of both clinical care and health system processes. Treatment success, death, treatment failure, and loss to follow-up represent conventional programme outcomes, while “not evaluated” outcomes are treated as potential signals of incomplete surveillance, weak patient tracking, or gaps in accountability for outcome assignment. Importantly, this interpretation is conceptual and framework-driven; the present study does not directly measure governance processes. Instead, it uses routine outcome patterns to explore whether incomplete reporting may indicate areas of system stress requiring further investigation [12]. This perspective shifts the analytical focus from outcome measurement alone to system-level diagnosis, enabling routine programme data to identify underlying weaknesses in surveillance and governance structures. Accordingly, this study aims to analyse longitudinal DR-TB treatment outcomes in rural Eastern Cape facilities between 2020 and 2024 and to examine whether patterns of incomplete outcome reporting, particularly “not evaluated” outcomes, can be interpreted as potential governance signals reflecting surveillance performance, data completeness, and accountability challenges.

## 2. Methods

### 2.1. Study Design and Data Source

This study employed a retrospective longitudinal descriptive analysis of aggregated DR-TB treatment outcomes over a five-year period (2020–2024). The design was intended to describe temporal patterns in treatment outcomes and surveillance completeness rather than evaluate causal relationships or directly measure governance and accountability processes. These facilities operate within a decentralized DR-TB care model in a predominantly rural setting characterized by high disease burden and resource constraints. The study population included all patients with bacteriologically confirmed rifampicin-resistant tuberculosis (RR-TB), encompassing multidrug-resistant TB (MDR-TB), pre-extensively drug-resistant TB (pre-XDR-TB), and extensively drug-resistant TB (XDR-TB), who were registered and initiated on treatment between January 2020 and December 2024. Patients without documented evidence of treatment initiation were excluded to ensure consistency in outcome assessment. Data were extracted from routinely maintained DR-TB treatment registers and the electronic DR-TB surveillance system (EDRWeb), which constitute standard data sources for provincial and national tuberculosis reporting in South Africa. To enhance data quality and reliability, a systematic cross-verification process was undertaken using facility-based DR-TB treatment registers, EDRWeb surveillance records, laboratory records, and district-level reporting summaries. Outcome counts and treatment category information were compared across sources to identify inconsistencies, missing entries, and potential duplicate records.

Where discrepancies were identified, the facility treatment register was treated as the primary source document and was cross-checked against surveillance and laboratory records to determine the most plausible value. Duplicate entries were reviewed and removed where records referred to the same treatment episode. Missing information was reconciled where corresponding information was available from an alternative source; otherwise, the original programme classification was retained.

Data quality review focused on consistency of treatment outcome assignment, completeness of annual outcome reporting, and agreement between facility and surveillance records. Because the study utilized routinely collected programme data and aggregated annual outcomes, formal inter-rater reliability testing was not applicable. The purpose of the verification process was to improve consistency and completeness of the dataset prior to descriptive analysis.

### 2.2. Study Setting

The study was conducted in two rural healthcare facilities located in the Eastern Cape Province of South Africa, a region characterized by a high burden of TB and DR-TB. These facilities operate within a decentralized model of DR-TB care, in which diagnosis, treatment initiation, and follow-up are delivered at the district and primary healthcare levels. The setting is marked by limited healthcare resources, including workforce shortages, constrained infrastructure, and challenges in patient tracking and data management. These contextual factors provide a relevant environment for examining both clinical outcomes and health system performance in DR-TB management.

The facilities serve predominantly rural and peri-rural populations within the Eastern Cape Province and provide decentralized DR-TB diagnosis, treatment initiation, monitoring, and follow-up services in accordance with national tuberculosis programme guidelines. The facilities receive referrals from surrounding primary healthcare clinics and district hospitals and manage patients with RR-TB, MDR-TB, pre-XDR-TB, and XDR-TB. The study setting is characterized by high levels of poverty, long travel distances to healthcare services, limited specialist support, and substantial TB-HIV burden, factors that may influence treatment continuity, patient retention, and surveillance performance.

The two study facilities were selected based on the availability and accessibility of routine DR-TB programme data and their role within the decentralized DR-TB care system. Facility selection was therefore purposive rather than representative and was not intended to provide a statistically representative sample of DR-TB treatment sites within the Eastern Cape Province or South Africa. Consequently, findings should be interpreted as reflecting the experiences and operational characteristics of these specific rural facilities rather than all DR-TB treatment settings.

### 2.3. Study Population

The study population comprised all patients with bacteriologically confirmed RR-TB, including MDR-TB, pre-XDR-TB, and XDR-TB, who were registered and initiated on treatment at the selected facilities during the study period (2020–2024). Inclusion of all resistance categories allowed for a comprehensive assessment of treatment outcomes across the DR-TB spectrum. Because the study population was restricted to patients managed within selected facilities, findings may reflect local operational, demographic, and health-system characteristics that differ from those of other DR-TB treatment sites.

### 2.4. Conceptual Framework: DR-TB Outcomes as Governance Signals

The conceptual framework underpins this study and represents its primary analytical contribution. It reconceptualizes DR-TB treatment outcomes as governance signals within a CE–CG approach, rather than viewing them solely as clinical endpoints. Importantly, the framework is not applied retrospectively; instead, it is embedded within the analytical process, shaping how routine programme data are interpreted. Within this perspective, outcomes such as treatment success, LTFU, and particularly those classified as ‘not evaluated’ are examined as programme indicators that may be informative regarding health system performance, accountability, and data integrity. However, these constructs were not directly measured in the present study. The CE–CG framework emphasizes that effective DR-TB care depends not only on clinical inputs but also on governance functions, including patient tracking systems, completeness and quality of data, and integration between facility-based and community health services. As such, longitudinal outcome trends observed in rural Eastern Cape facilities are interpreted as indicators of system capacity and data management strength, rather than clinical effectiveness alone. This approach shifts the analytical focus from outcome measurement to system-level diagnosis.

The CE–CG framework was used as an interpretive lens for understanding observed outcome patterns. However, governance structures, accountability mechanisms, patient-tracing systems, and surveillance processes were not directly measured within the study and therefore cannot be empirically confirmed as explanations for the observed trends.

**Figure 1:**
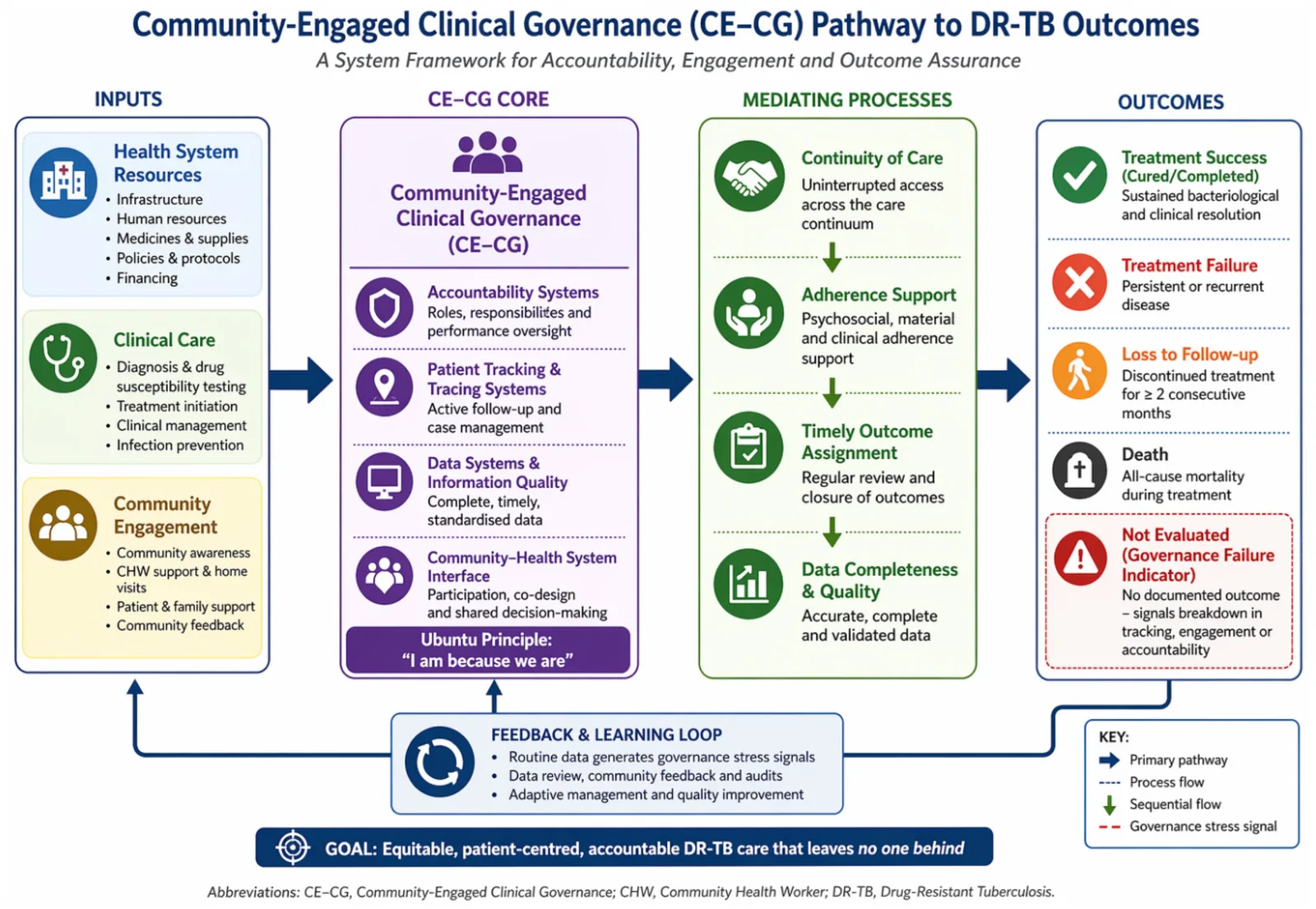
The framework illustrates how health system resources, clinical care, and community engagement interact through governance processes to influence continuity of care, adherence, outcome assignment, and data quality. Outcomes are interpreted as both clinical indicators and governance signals, with “not evaluated” representing a potential indicator of weaknesses in accountability and surveillance systems. A feedback loop enables continuous system learning and improvement.

As illustrated in Figure 1, the framework adopts a systems-based perspective linking inputs, governance processes, and programme outcomes. At the input level, three foundational domains are integrated:

- Health system resources (infrastructure, workforce, medicines, and financing).
- Clinical care processes (diagnosis, treatment initiation, and infection control).
- Community engagement (community health workers, patient support systems, and participation).

These domains converge within the core of the CE–CG model, which operationalizes governance through accountability systems, patient tracking and tracing mechanisms, data systems and information quality, and a strengthened community–health system interface. Guided by the Ubuntu principle *“I am because we are,”* the framework emphasizes that effective DR-TB management is inherently relational and participatory. Through key mediating processes including continuity of care, adherence support, timely outcome assignment, and data completeness, governance functions are translated into measurable programme outcomes, shaping both patient care and surveillance integrity. At the outcome level, the model distinguishes between conventional clinical outcomes (treatment success, failure, LTFU, and death) and those classified as “not evaluated” outcomes are explicitly reframed as potential indicators of governance-related challenges, surveillance limitations, or accountability gaps that warrant further investigation. This reconceptualization represents a central contribution of the study, positioning incomplete outcomes as signals of possible accountability gaps, patient tracking, and system performance. Finally, the framework incorporates feedback and a learning loop, highlighting the dynamic role of routine data in generating governance “stress signals,” informing programme audits, supporting community feedback, and enabling adaptive improvements in health system performance.

#### Operational Interpretation of Governance Signals

Governance, accountability, and surveillance integrity were not directly measured as study variables. No specific governance indicators, accountability metrics, or health-system performance measures were collected or analyzed. Instead, the CE–CG framework was applied as an interpretive framework for understanding routine programme outcomes. Within this framework, the term “governance signals” refers to observable programme indicators that may reflect the functioning of surveillance and accountability processes. Specifically, increasing proportions of outcomes classified as “not evaluated” and declining outcome completeness were interpreted as potential indicators of challenges in patient tracking, outcome assignment, reporting systems, and surveillance performance. These indicators were not treated as direct measures of governance but rather as programme-level patterns that may warrant further investigation of underlying governance and accountability processes. Accordingly, governance-related interpretations presented in this study should be regarded as conceptual and hypothesis-generating rather than empirical measurements of governance capacity or accountability performance.

### 2.5. Outcome Definitions

Treatment outcomes were defined according to standard national and World Health Organization (WHO) guidelines for drug-resistant tuberculosis (DR-TB). Outcomes included: treatment success (cured or treatment completed), treatment failure, loss to follow-up (LTFU), death, and not evaluated. The “not evaluated” category included patients for whom treatment outcomes were not assigned at the time of reporting due to incomplete follow-up, missing documentation, or unresolved treatment status.

### 2.6. Statistical Analysis

Descriptive analyses were conducted to summarise treatment outcomes across resistance categories and study years. Annual outcome proportions were calculated with corresponding 95% confidence intervals using the Wilson method, which provides more robust interval estimation for binomial proportions, particularly in small-sample and sparse-data settings. To assess temporal changes in outcome distributions, chi-square tests of independence were applied to RR-TB and MDR-TB categories, where sample sizes were sufficient to support exploratory comparisons. For pre-XDR-TB and XDR-TB categories, inferential testing was retained for completeness but interpreted with extreme caution because of very small cell counts and limited statistical power. Consequently, analyses for these subgroups were considered primarily descriptive and exploratory, with greater emphasis placed on observed patterns, confidence intervals, and outcome completeness than on statistical significance. The inferential analyses were intended to identify potential shifts in outcome distributions rather than establish definitive differences or causal relationships. Statistical significance was defined at a threshold of *p* < 0.05.

Formal trend tests were not employed because outcome patterns were not assumed to be linear or monotonic across the five study years, and substantial fluctuations in outcome completeness were observed over time. Regression modelling was not undertaken because the dataset consisted of aggregated annual outcome counts rather than patient-level observations, limiting adjustment for potential confounding variables and increasing the risk of ecological inference. Interrupted time-series analysis was also considered inappropriate because the study did not evaluate a clearly defined intervention, policy change, or implementation event occurring at a specific time point, and the availability of only five annual observations would not support robust time-series modelling.

### 2.7. Handling of Missing Data and Bounding Analysis

Given the increasing proportion of outcomes classified as “not evaluated,” additional analyses were undertaken to assess the impact of incomplete outcome reporting on key programme indicators. In this context, missing outcome data were considered likely to be missing not at random (MNAR), as the absence of outcome assignment may be associated with underlying patient-level factors (e.g., loss to follow-up, undocumented mortality) or system-level factors (e.g., weaknesses in patient tracing, delayed reporting, or data management limitations). To address this, bounding analyses were conducted to evaluate the sensitivity of treatment success estimates to alternative assumptions regarding cases classified as “not evaluated.” Specifically, two extreme scenarios were considered: (1) a best-case scenario in which all unevaluated cases were assumed to have favorable outcomes (i.e., treatment success), and (2) a worst-case scenario in which all unevaluated cases were assumed to have unfavorable outcomes (i.e., failure, death, or LTFU). These bounds provide a plausible range for the true treatment success rate. This approach does not attempt to impute missing data but instead quantifies the uncertainty introduced by incomplete outcome ascertainment. By explicitly incorporating uncertainty due to MNAR mechanisms, bounding analysis supports more cautious and transparent interpretation of routine programme indicators, particularly in settings where data completeness is limited and standard missing-data assumptions are unlikely to hold.

## 3. Results

### 3.1. Patient Characteristics and Aggregate Treatment Outcomes

A total of 402 patients with DR-TB were included across all resistance categories between 2020 and 2024. RR-TB and MDR-TB accounted for the majority of cases, representing 47.5% (*n* = 191) and 44.3% (*n* = 178), respectively. In contrast, pre-XDR-TB; 5.7%, (*n* = 23) and XDR-TB; 2.5%, (*n* = 10) constituted relatively small subgroups. Treatment outcomes varied across resistance categories. Overall treatment success was highest among pre-XDR-TB cases (73.9%, *n* = 17/23), followed by RR-TB (68.1%, *n* = 130/191), MDR-TB (67.4%, *n* = 120/178), and XDR-TB (60.0%, *n* = 6/10). Unfavourable outcomes, including treatment failure, LTFU, and death, were most pronounced among MDR-TB and RR-TB cases. Mortality was highest in RR-TB (16.2%, *n* = 31/191), followed by MDR-TB (8.4%, n = 15/178), while LTFU was also substantial in these groups (RR-TB: 11.0%; MDR-TB: 10.1%). Outcomes classified as “not evaluated” were observed across multiple resistance categories and represent an important source of uncertainty in outcome interpretation. The proportion of unevaluated outcomes was highest in XDR-TB (30%, *n* = 3/10), followed by MDR-TB (6.7%, *n* = 12/178) and RR-TB (4.7%, *n* = 9/191), while no such cases were observed in the pre-XDR-TB group. Although these proportions are modest in aggregate, subsequent analyses demonstrate that “not evaluated” outcomes increased over time, particularly in later years, and may substantially influence the interpretation of programme performance. Collectively, these findings highlight a dual pattern of clinical and system-level concern: while unfavorable outcomes remain substantial in high-burden resistance categories, incomplete outcome ascertainment introduces additional uncertainty and may obscure true treatment performance Table 1.

Because the study relied on aggregated routine programme data, detailed patient-level characteristics such as age, sex, HIV status, previous TB treatment history, socioeconomic status, and comorbid conditions were not available for analysis. Consequently, the study focuses on programme-level outcome patterns rather than individual-level predictors of treatment success or failure.

### 3.2. RR-TB Trends over Time

RR-TB treatment outcomes demonstrated temporal variability across the study period (2020–2024). Treatment success increased from 71.0% (95% CI: 53.4–83.9) in 2020 to a peak of 79.5% (65.5–88.8) in 2021, followed by a decline to 61.2% (47.2–73.6) in 2022, a partial recovery in 2023 (75.0%; 58.9–86.2), and a marked decrease to 51.6% (34.8–68.0) in 2024. Although the overall decline in treatment success did not reach statistical significance (*p* = 0.075), the wide and overlapping confidence intervals indicate substantial uncertainty and limited precision due to relatively small sample sizes. Importantly, the decline in observed treatment success coincided with a significant increase in outcomes classified as “not evaluated,” which rose from 0% in 2020–2022 to 2.8% in 2023 and sharply to 25.8% in 2024 (*p* < 0.001). This pattern suggests that the apparent reduction in treatment success in later years may be partly attributable to incomplete outcome ascertainment rather than a true deterioration in clinical outcomes. Mortality remained consistently elevated throughout the study period, ranging from 8.3% to 25.8%, indicating a persistent burden of severe disease or delayed treatment initiation. LTFU fluctuated, peaking at 18.4% in 2022 before declining to 9.7% in 2024. The reduction in LTFU in later years, alongside the rise in “not evaluated” outcomes, raises the possibility of outcome misclassification or incomplete follow-up. Overall, these findings highlight a key system-level pattern: shifts in outcome completeness appear to strongly influence observed trends in treatment success. This reinforces the importance of interpreting routine RR-TB outcomes within the context of data quality and surveillance system performance, rather than attributing changes solely to clinical factors as indicated in Table 2.

**Figure 2:**
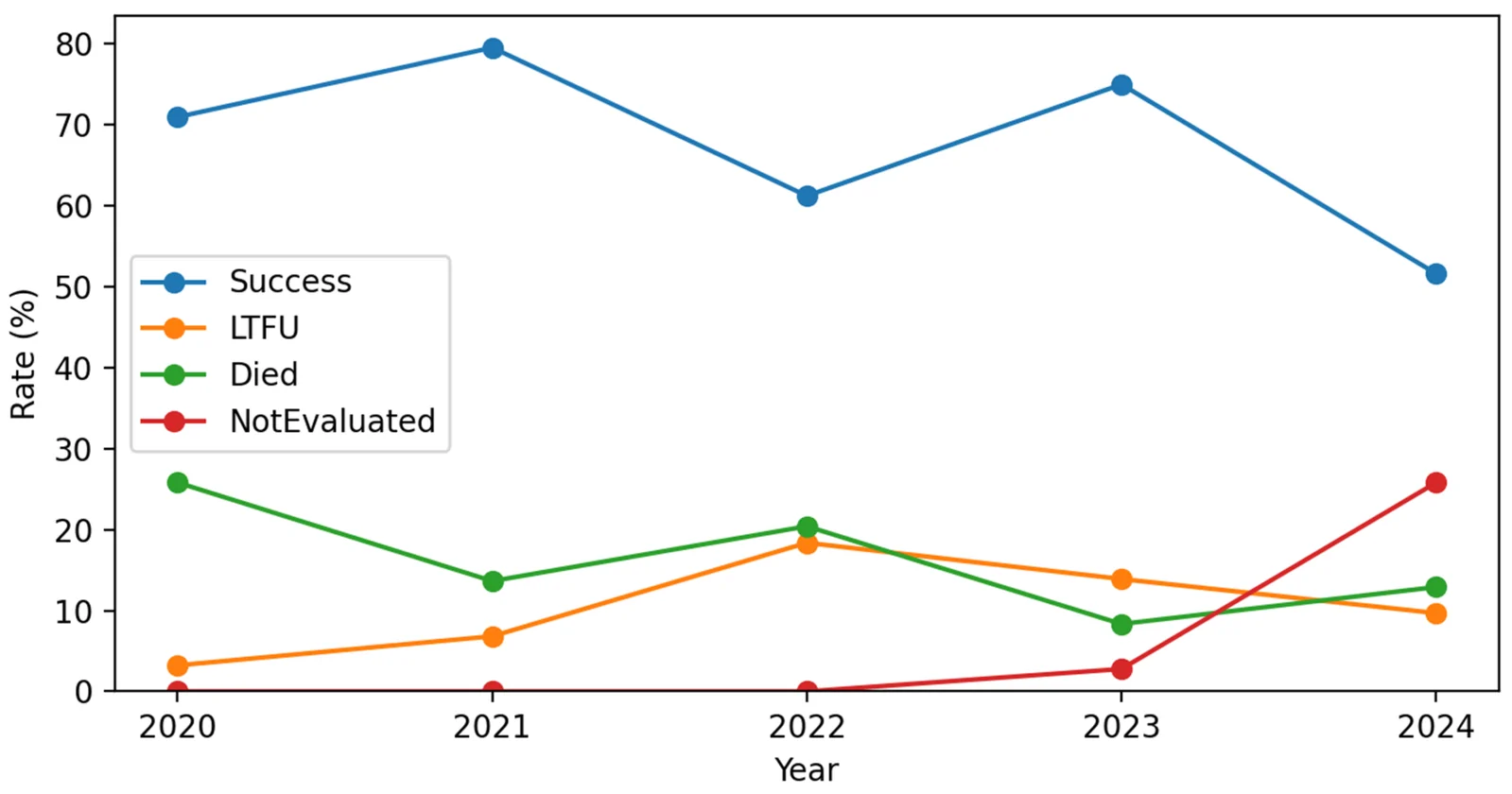
RR-TB outcome rates by year (2020–2024).

Figure 2 illustrates temporal trends in rifampicin-resistant tuberculosis (RR-TB) treatment outcomes between 2020 and 2024, highlighting variability in treatment success alongside shifts in other outcome categories. Treatment success initially improved from 2020 to 2021, reaching a peak in 2021, then declined in 2022, partially recovered in 2023, and reached a marked reduction in 2024. However, this decline in success is accompanied by a pronounced increase in outcomes classified as “not evaluated,” particularly in 2024, where it rises sharply relative to previous years. Mortality remains consistently high across the study period, although it shows a slight downward trend after 2022. LTFU peaks in 2022 and subsequently declines, suggesting possible improvements in patient retention. Notably, the reduction in LTFU in later years coincides with the emergence and rapid increase of “not evaluated” outcomes. This pattern suggests that the apparent decline in treatment success in 2024 may be due to incomplete outcome ascertainment rather than a true deterioration in clinical performance. The inverse relationship between “not evaluated” outcomes and both treatment success and LTFU suggests potential misclassification of outcomes or gaps in patient tracking and reporting systems. Overall, the figure highlights a key system-level signal: increasing data incompleteness appears to distort observed outcome trends, reinforcing the need to interpret RR-TB programme performance within the context of surveillance quality and health system governance.

### 3.3. MDR-TB Trends over Time

Table 3 Shows that MDR-TB demonstrated the most pronounced temporal variation in treatment outcomes over the study period (2020–2024). Treatment success increased from 67.6% (95% CI: 50.8–80.9) in 2020 to a peak of 80.9% (67.5–89.6) in 2021, followed by a decline to 66.7% (51.0–79.4) in 2022 and 71.9% (54.6–84.4) in 2023, before sharply decreasing to 38.5% (22.4–57.5) in 2024. This decline was statistically significant (*p* = 0.007). However, the wide and overlapping confidence intervals across years indicate substantial uncertainty and reduced precision, particularly in later years with smaller sample sizes. The observed decline in treatment success coincided with a marked increase in outcomes classified as “not evaluated,” which rose from 0% in 2020–2022 to 12.5% in 2023 and further to 30.8% in 2024 (*p* < 0.001). This temporal co-movement strongly suggests that the apparent deterioration in treatment success is influenced, at least in part, by incomplete outcome ascertainment rather than representing a purely clinical decline. Failure rates decreased over time, reaching 0% in 2024. However, this pattern should be interpreted cautiously, as it may reflect reclassification or displacement of outcomes into the “not evaluated” category rather than a true reduction in treatment failure. Mortality peaked in 2022 (17.9%) before declining in subsequent years, while LTFU fluctuated moderately without a clear directional trend. Importantly, bounding analyses (see Section 2) indicate that treatment success estimates in later years are highly sensitive to assumptions regarding missing outcomes. Under plausible scenarios, reclassifying “not evaluated” cases could substantially alter estimated success rates, highlighting the extent of uncertainty introduced by incomplete reporting.

Overall, these findings underscore a critical system-level insight: the sharp decline in MDR-TB treatment success in 2024 is may be associated with increasing data incompleteness. This reinforces the interpretation that observed trends reflect not only clinical outcomes but also underlying weaknesses in surveillance, patient tracking, and outcome assignment processes.

**Figure 3:**
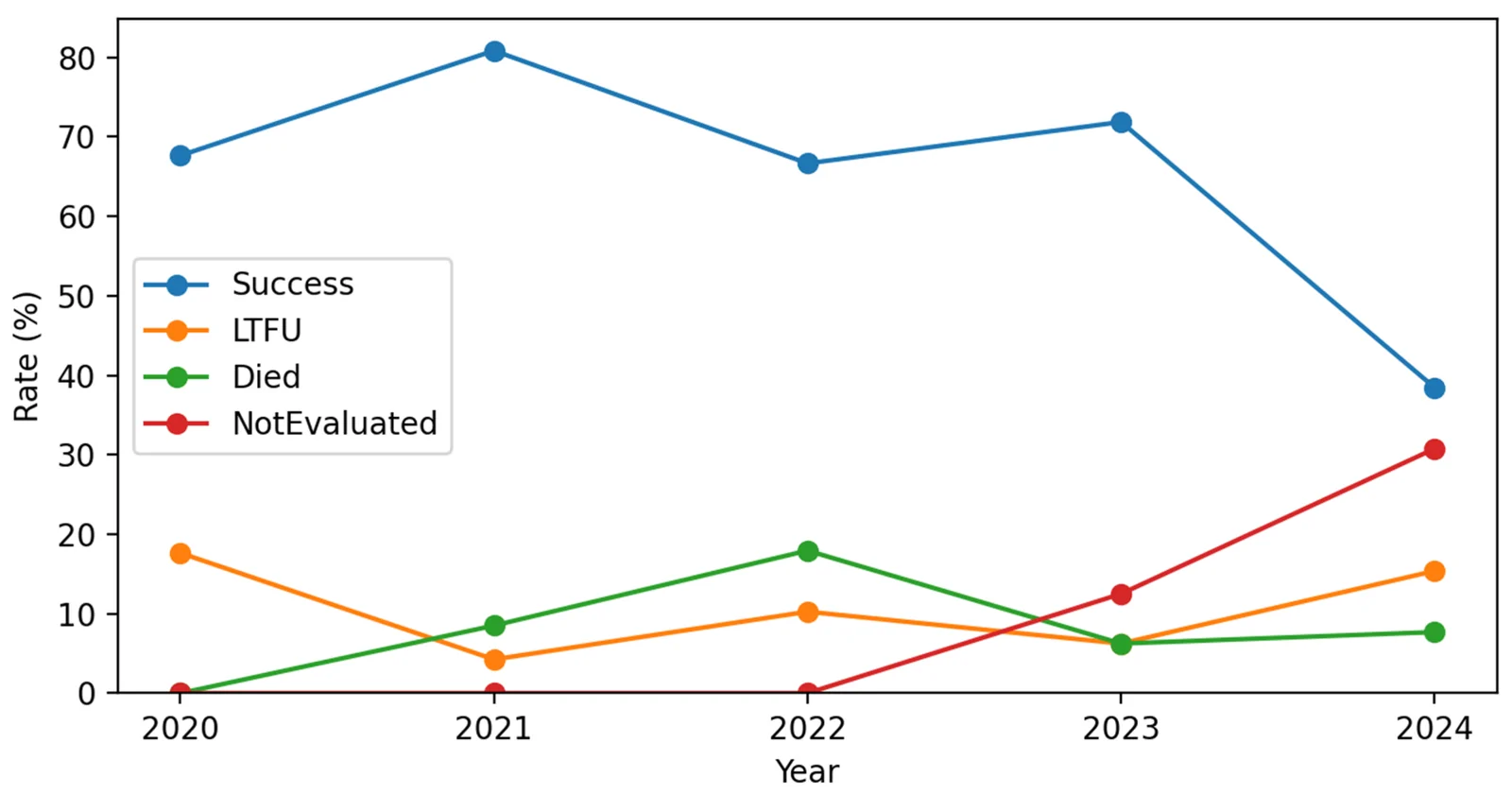
MDR-TB outcome rates by year (2020–2024).

Figure 3 presents temporal trends in MDR-TB treatment outcomes from 2020 to 2024, highlighting substantial variability across outcome categories and a pronounced shift in the composition of outcomes over time. Treatment success improved from 2020 to a peak in 2021, followed by moderate fluctuations in 2022 and 2023, and a sharp decline in 2024. This decline coincides with a marked rise in outcomes classified as “not evaluated,” which emerge in 2023 and increase substantially in 2024. The inverse relationship between treatment success and “not evaluated” outcomes suggests that the observed reduction in success rates in later years may be partly attributable to incomplete outcome ascertainment rather than a true deterioration in clinical performance. Treatment failure shows a consistent decline over the study period, reaching zero in 2024. However, this pattern should be interpreted cautiously, as it may reflect reclassification or displacement of outcomes into the “not evaluated” category rather than a genuine reduction in failure rates. Mortality peaks in 2022 and subsequently declines, while LTFU fluctuates without a clear trend, indicating variability in patient retention and follow-up processes. Overall, the figure suggests a key system-level pattern: increasing data incompleteness in later years appears to significantly influence observed outcome distributions. The rise in “not evaluated” outcomes likely reflects underlying challenges in patient tracking, outcome assignment, and data system performance. These findings reinforce the importance of interpreting MDR-TB programme outcomes within a broader governance and surveillance context, rather than attributing changes solely to clinical factors.

### 3.4. Pre-XDR-TB Outcomes over Time

Treatment outcome estimates for pre-extensively pre-XDR-TB were characterized by substantial statistical uncertainty due to very small sample sizes across all study years (*n* = 3–9 per year) (Table 4). As a result, findings should be interpreted with caution, and emphasis should be placed on descriptive patterns rather than inferential conclusions. Given the annual sample sizes of only 3–9 cases, statistical comparisons have limited interpretive value and should not be considered evidence of temporal stability or change. Consequently, descriptive interpretation of trends is more appropriate than reliance on *p*-values for this subgroup. Across the study period, treatment success remained relatively stable, ranging from 66.7% to 80%, with no statistically significant variation over time (*p* = 0.982). However, the wide and overlapping 95% confidence intervals, for example, 20.8–93.9% in multiple years, indicate low precision and limited reliability of point estimates. These wide intervals reflect the small denominators and suggest that observed differences are likely due to random variation rather than meaningful changes in programme performance. Other outcome categories showed sporadic variation without consistent temporal trends. Mortality was observed intermittently, reaching 33.3% in several years, while treatment failure LTFU were infrequent and inconsistent. Notably, no outcomes were classified as “not evaluated” during the study period for this subgroup, suggesting relatively complete outcome ascertainment compared with the RR-TB and MDR-TB categories. Overall, the findings for pre-XDR-TB are best interpreted as descriptive and exploratory. The absence of clear temporal trends, combined with wide confidence intervals, limits the ability to draw firm conclusions regarding treatment performance or system-level influences in this subgroup.

**Figure 4:**
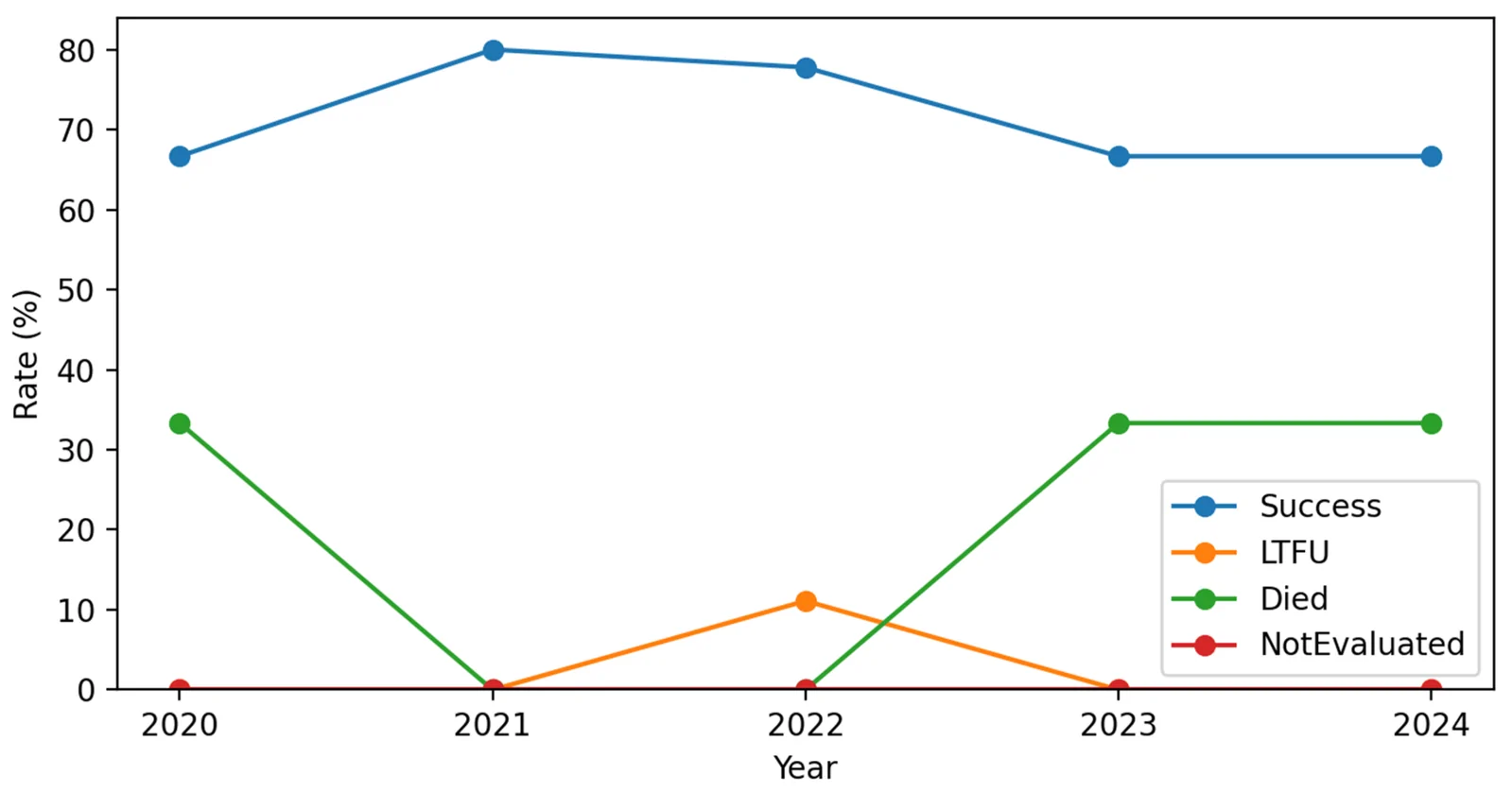
Pre XDR -TB outcome rates by year (2020–2024).

Figure 4 shows that treatment outcomes for pre-XDR-TB remained relatively stable over the study period, with treatment success consistently ranging between approximately 66.7% and 80%. There is no clear trend in treatment success, and the wide confidence intervals indicate substantial statistical uncertainty due to very small sample sizes. Variations in mortality, including peaks of 33.3% in some years, appear intermittent and inconsistent, suggesting random fluctuation rather than systematic changes in programme performance. Similarly, treatment failure and LTFU occur infrequently and without a consistent pattern. Importantly, no outcomes were classified as “not evaluated” across all years, indicating relatively complete outcome ascertainment in this subgroup. This contrasts with trends in RR-TB and MDR-TB, where increasing incomplete reporting influenced observed outcomes. The figure suggests that pre-XDR-TB outcomes are relatively stable, but the small number of cases limits interpretability. Observed variations are likely driven by low sample sizes rather than meaningful changes in clinical outcomes or health system performance.

### 3.5. XDR-TB Outcomes over Time

Table 5 Treatment outcomes for extensively drug-resistant tuberculosis (XDR-TB) exhibited substantial variability across the study period (2020–2024); however, interpretation is severely limited by extremely small sample sizes (*n* = 1–3 per year). As a result, findings should be considered descriptive and exploratory rather than indicative of underlying programme trends. Because annual case numbers ranged from one to three patients, inferential statistics in this subgroup are highly unstable and should be interpreted as exploratory only. Treatment success was reported as 100% in 2020 and 2021, declining to 66.7% (95% CI: 20.8–93.9) in 2022, and further to 0% in both 2023 and 2024. Despite this apparent decline, the wide confidence intervals and very small denominators indicate high statistical uncertainty, and these changes should not be interpreted as evidence of a true deterioration in clinical outcomes. Notably, outcomes classified as “not evaluated” increased from 0% in 2020–2022 to 100% in both 2023 and 2024 (*p* = 0.040). While this shift reached statistical significance, it is driven by extremely small numbers and should be interpreted with caution. The emergence of unevaluated outcomes in this subgroup may nonetheless indicate potential gaps in patient follow-up, outcome assignment, or surveillance processes, particularly in managing patients with the most severe forms of drug resistance. Other outcome categories, including treatment failure, LTFU, and death, were infrequent and showed no consistent temporal pattern. The absence of deaths across all years may reflect a limited sample size rather than the true absence of mortality. XDR-TB outcome patterns are dominated by statistical instability and limited interpretability. However, the appearance of “not evaluated” outcomes in later years aligns with broader system-level trends observed in RR-TB and MDR-TB, suggesting that data completeness and surveillance capacity may influence outcome reporting even in small, high-risk subgroups.

**Figure 5:**
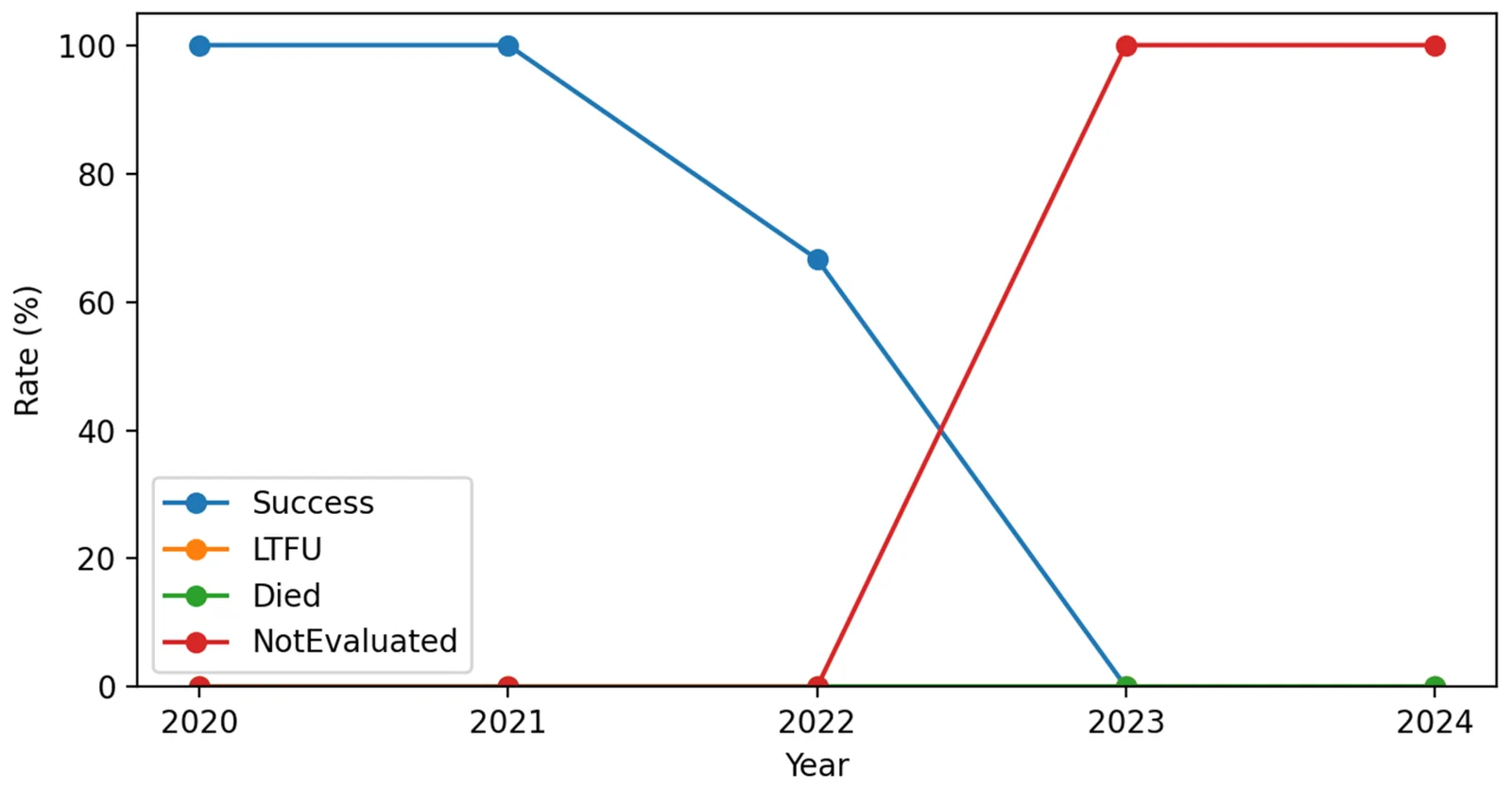
Treatment outcomes among patients with extensively drug-resistant tuberculosis (XDR-TB) by year, 2020–2024. Annual proportions of treatment outcomes for patients diagnosed with XDR-TB from 2020 to 2024. The figure illustrates temporal trends in treatment success, mortality, loss to follow-up, treatment failure, and other recorded outcomes. Colours in the legend correspond to the categories displayed in the figure.

Figure 5 illustrates substantial year-to-year variability in treatment outcomes for XDR-TB; however, interpretation is constrained by extremely small sample sizes. Treatment success appears high in the early years (2020–2021), declines in 2022, and drops to 0% in 2023–2024. Despite this apparent downward trend, the very wide confidence intervals and the small number of cases indicate that these fluctuations are not reliable indicators of true changes in clinical performance. A key feature of the figure is the emergence of outcomes classified as “not evaluated” in 2023 and 2024, reaching 100% in both years. This shift suggests a breakdown in outcome ascertainment, likely reflecting gaps in patient follow-up, reporting, or data management rather than a true absence of successful treatment outcomes. Other outcome categories, including failure, LTFU, and death, show minimal variation and remain infrequent, though this may be influenced by small denominators. The figure highlights that XDR-TB outcome patterns are dominated by statistical instability. However, the rise in “not evaluated” outcomes aligns with trends observed in RR-TB and MDR-TB, reinforcing the interpretation that data completeness and surveillance capacity are key determinants of observed programme performance.

### 3.6. System-Level Patterns

The system-level trends in treatment success and surveillance completeness across drug-resistant tuberculosis (DR-TB) categories are summarized in Table 6. Across the 2020–2024 study period, a consistent system-level pattern emerged in which increasing proportions of outcomes classified as “not evaluated” coincided with apparent declines in treatment success, particularly among rifampicin-resistant tuberculosis (RR-TB) and multidrug-resistant tuberculosis (MDR-TB) cases. This co-occurrence suggests that observed outcome trends are shaped not only by clinical performance but also by the completeness of underlying data, surveillance functionality, and health system governance capacity. The rise in “not evaluated” outcomes represents a dominant programmatic signal that became increasingly pronounced from 2023 onwards.

As shown in Table 6, apparent declines in treatment success were observed, most notably for MDR-TB. These patterns indicate that treatment success estimates in the later years are highly sensitive to incomplete outcome ascertainment, reinforcing concerns regarding the reliability and interpretation of routine programme performance indicators. For MDR-TB, treatment success declined significantly from 80.9% in 2021 to 38.5% in 2024 (*p* = 0.007), paralleling a marked increase in “not evaluated” outcomes from 0% to 30.8% (*p* < 0.001). This temporal co-occurrence suggests that the apparent deterioration in treatment success is likely influenced not only by clinical factors but also by incomplete outcome reporting, which may distort programme performance estimates. A similar pattern was observed for RR-TB. Although treatment success declined from 79.5% in 2021 to 51.6% in 2024, the difference was not statistically significant (*p* = 0.075). However, the increase in “not evaluated” outcomes from 0% during 2020–2022 to 25.8% in 2024 was highly significant (*p* < 0.001) (Table 6). This divergence further supports the interpretation that incomplete outcome reporting, rather than true clinical deterioration alone, may explain much of the observed decline in programme performance. In contrast, treatment outcomes among patients with pre-extensively drug-resistant tuberculosis (pre-XDR-TB) remained relatively stable throughout the study period (66.7–80%; *p* = 0.982). However, this apparent stability should be interpreted with caution given the small sample sizes and limited statistical power. Similarly, among patients with extensively drug-resistant tuberculosis (XDR-TB), “not evaluated” outcomes increased from 0% during 2020–2022 to 100% in 2023–2024 (*p* = 0.040) (Table 6). Although interpretation is constrained by the very small number of XDR-TB cases, the emergence of incomplete outcomes in this high-risk group may indicate deficiencies in patient follow-up, outcome ascertainment, or surveillance processes. Overall, these findings demonstrate a consistent pattern across DR-TB categories whereby apparent declines in treatment success were accompanied by increasing proportions of outcomes classified as “not evaluated”. Within the Community Engagement–Clinical Governance (CE-CG) framework, this pattern may represent a governance signal consistent with weaknesses in patient tracking, outcome reporting, data completeness, and surveillance performance. However, these mechanisms were not directly evaluated in the present study. Consequently, routine DR-TB treatment outcomes should be interpreted not only as measures of clinical performance but also as indicators of health system functionality and surveillance integrity.

### 3.7. Summary of Key Findings

Across all resistance categories, the most salient pattern is not variation in mortality or LTFU, but the progressive increase in outcomes classified as “not evaluated.” This trend is most pronounced in RR-TB and MDR-TB and temporally coincides with apparent declines in treatment success in later years. The consistent co-movement between increasing “not evaluated” proportions and decreasing success rates indicates that observed outcome trends are strongly influenced by incomplete outcome ascertainment. While mortality remained persistently high in RR-TB and showed temporal fluctuations in MDR-TB, these patterns were less influential in shaping overall outcome distributions than the impact of missing or unassigned outcomes. This suggests that data incompleteness may have a greater effect on reported program performance than changes in clinical outcomes. In contrast, findings for pre-XDR-TB and XDR-TB remain descriptive and should be interpreted with caution due to small sample sizes, wide confidence intervals, and limited statistical power. As such, no reliable temporal trends can be inferred for these subgroups.

Overall, the results highlight a critical methodological and programmatic insight: substantial increases in data incompleteness substantially limit the interpretability of routine DR-TB outcome indicators. Within a governance-oriented framework, the rising proportion of “not evaluated” outcomes can be interpreted as a system-level signal of weaknesses in patient tracking, outcome assignment, and surveillance processes, rather than as a neutral category of missing data.

## 4. Discussion

This study identified temporal changes in DR-TB treatment outcomes and outcome completeness within a rural Eastern Cape setting. The observed increase in outcomes classified as “not evaluated” occurred alongside declining reported treatment success, particularly among RR-TB and MDR-TB patients. While these patterns are consistent with potential weaknesses in surveillance and accountability systems, the present study was not designed to directly evaluate governance processes or determine causal explanations for the observed trends. By integrating statistical uncertainty with a governance-oriented analytical framework, this study advances a system-level interpretation of routine DR-TB outcome data, highlighting the co-production of program indicators through both clinical care and data management processes. In this high-burden rural setting, the distribution of RR-TB and MDR-TB reflects a sustained burden of resistant disease with implications for ongoing transmission and program effectiveness. The application of Wilson confidence intervals underscores the limited precision of outcome estimates, particularly in subgroups with small sample sizes. It reveals that apparent differences in treatment success may encompass a wide range of plausible underlying values. This statistical uncertainty constrains the strength of inferences that can be drawn from routine data and highlights the importance of cautious interpretation. Inferential analyses indicate that mortality is the only outcome consistently demonstrating detectable variation. In contrast, other outcome categories including treatment success, failure, and loss to follow-up are highly sensitive to sample size, variability, and data completeness. Notably, the increasing proportion of outcomes classified as “not evaluated” is a critical system-level signal, reflecting potential breakdowns in patient follow-up, outcome assignment, and data reporting. These gaps may obscure the true burden of disease and distort estimates of program performance, particularly in decentralized and resource-constrained settings. Collectively, these findings reinforce the central premise of the CE–CG framework: routine DR-TB outcome indicators should not be interpreted solely as measures of clinical effectiveness but also as reflections of health system capacity, surveillance integrity, and accountability mechanisms. As such, trends in treatment outcomes must be contextualized within broader system dynamics, including data completeness, patient tracking, and the functionality of routine surveillance systems, to enable more accurate assessment of program performance and transmission risk.

### 4.1. Burden, Transmission Dynamics, and Programmatic Severity

The distribution of DR-TB cases across resistance categories highlights a substantial burden of disease within the study setting, with RR-TB and MDR-TB accounting for the majority of cases. This pattern is consistent with high-burden contexts where rifampicin resistance serves as a key proxy for broader drug resistance and ongoing community-level transmission [11]. The predominance of these categories suggests that transmission of resistant strains, rather than de novo emergence during treatment, remains a primary driver of the local epidemic. In South Africa, molecular surveillance studies have demonstrated substantial clustering of RR-TB and MDR-TB strains across provinces, indicating recent transmission rather than independent acquisition events [17]. National genomic analyses further estimate that a significant proportion of RR-TB cases are attributable to recent transmission, underscoring the importance of community-level spread in sustaining the epidemic [13,17]. Local evidence from the Eastern Cape aligns with these findings. Facility-based studies in rural districts have documented geographically concentrated burdens of RR-TB and MDR-TB, alongside delayed diagnosis, prolonged treatment pathways, and constrained service capacity. Although molecular genotyping was not performed in these settings, the observed spatial and programmatic patterns are epidemiologically consistent with ongoing local transmission rather than widespread de novo resistance emergence [16]. Program-based analyses further indicate that delayed diagnosis, prolonged infectiousness, and systemic constraints within decentralized models of care contribute to sustained DR-TB transmission, particularly among economically active populations [12]. Collectively, these findings reinforce that the observed RR-TB and MDR-TB patterns reflect transmission-driven dynamics typical of high-burden low- and middle-income country (LMIC) settings. From a prevalence perspective, the sustained presence of RR-TB and MDR-TB across all study years indicates persistent community-level exposure to resistant *Mycobacterium tuberculosis*. In such contexts, transmission is likely to be amplified by delayed diagnosis, incomplete treatment, and weak contact-tracing systems. These findings are consistent with studies conducted in India, Germany, and Ethiopia, which highlight disease progression driven by delayed treatment access, poor TB knowledge, and inadequate contact tracing strategies [18,19,20]. Additionally, research from Cape Town has demonstrated that stigma, resistance to screening, limited health literacy, and the need for culturally sensitive interventions contribute to ongoing transmission of drug-resistant TB. Structural factors—including resource constraints, overburdened healthcare systems, and weak public health messaging—further exacerbate these dynamics [21]. In rural settings, limited access to diagnostic services and delays in treatment initiation may prolong infectious periods, thereby increasing transmission risk [11]. Observed patterns of mortality and disease progression, particularly among RR-TB and MDR-TB patients, further underscore the implications for transmission control. Patients who are LTFU or inadequately monitored may remain infectious, contributing to sustained community transmission [22,23]. Similarly, elevated mortality may reflect late presentation or advanced disease, both of which signal missed opportunities for early diagnosis and interruption of transmission chains [24]. The most concerning programmatic signal, however, is the rising proportion of outcomes classified as “not evaluated,” particularly in later years. While not a biological endpoint, this category represents a critical gap in surveillance and patient management. From a transmission perspective, “not evaluated” cases may conceal individuals who remain untreated, inadequately treated, or untracked within the health system, thereby constituting a potentially unmeasured reservoir of ongoing transmission [25].

In terms of program severity, MDR-TB emerges as the most critical category in this study. The combination of declining apparent treatment success, increasing incomplete outcome reporting, and the inherent complexity of MDR-TB treatment suggests heightened vulnerability to both clinical and system-level failures. Importantly, the rise in “not evaluated” outcomes indicates that the most significant programmatic risk may not be treatment failure alone, but the health system’s inability to account for patient outcomes. This reframes “severity” from a purely clinical construct to a governance-informed concept in which system invisibility becomes a key risk factor. Although pre-XDR-TB and XDR-TB cases were limited in number, their presence remains epidemiologically significant. These forms of resistance are associated with restricted treatment options, prolonged infectiousness, and a higher likelihood of adverse outcomes. Even at low prevalence, they represent potential amplification points for the transmission of highly resistant strains if not effectively contained [2,8,26]. Taken together, these findings indicate that the burden of DR-TB in this setting is shaped not only by disease prevalence but also by the interaction between transmission dynamics and health system performance. The most critical threat to TB control is therefore not solely the presence of resistant disease, but the convergence of ongoing transmission, incomplete patient follow-up, and weak surveillance systems. Strengthening early diagnosis, improving treatment continuity, and ensuring complete outcome ascertainment are essential not only for individual patient care but also for interrupting transmission and reducing overall disease burden. The marked increase in outcomes classified as “not evaluated,” particularly from 2023 onward, should not be interpreted as a neutral reporting artifact. Consistent with global tuberculosis surveillance guidance, treatment outcome completeness is a core dimension of surveillance quality and program governance, reflecting the strength of accountability mechanisms linking clinical service delivery to health system oversight [27]. The World Health Organization similarly emphasizes that complete outcome reporting is essential for valid interpretation of treatment success trends, and that rising levels of missing outcomes may signal breakdowns in patient tracking, responsibility assignment, and information system integration [28]. Alternative explanations—including patient mobility, undocumented deaths outside the formal health system, inter-facility transfers, and delays in data capture—should also be considered when interpreting incomplete treatment outcomes in routine datasets. Patient mobility and undocumented mortality are increasingly recognized as key drivers of incomplete outcome reporting in high-burden settings [29]. The WHO global tuberculosis report highlights that loss to follow-up and unreported outcomes are structurally linked to population mobility, fragmented referral systems, and weak inter-facility information exchange [30]. For example, patients may initiate treatment in one district and continue care in another without formal documentation (“silent transfers”), leading to misclassification as treatment interruption or “not evaluated” in routine datasets [31]. Additionally, mortality may be underestimated due to undocumented deaths occurring outside health facilities, particularly among patients disengaged from care. Weak linkage between TB registers and electronic death registration systems further contributes to incomplete mortality ascertainment [30]. Evidence from TB surveillance evaluations in high-burden countries shows that some patients initially classified as LTFU are later found to have died or transferred without documentation, indicating that routine datasets may underestimate both mortality and disease burden when tracing systems are weak [4,30].

Within this context, the observed escalation of “not evaluated” outcomes in rural Eastern Cape facilities is more plausibly interpreted as evidence of underlying surveillance and governance weaknesses rather than a purely analytical limitation. This distinction is critical, as governance-related data gaps can distort program performance indicators and lead to misleading interpretations of declining clinical effectiveness. Evidence from decentralized DR-TB programs in South Africa suggests that expanding access without concurrent strengthening of clinical governance and information systems can compromise both care quality and reporting reliability [14,27]. These findings reinforce the idea that observed program outcomes reflect not only clinical realities but also the performance of the underlying surveillance and governance systems.

### 4.2. Structural Rural Health-System Constraints and Outcome Ascertainment

Elevated RR-TB mortality observed in this study may reflect multiple interacting factors, including delayed diagnosis, advanced disease at treatment initiation, co-morbidity burden, and limitations in clinical stabilization capacity. However, in the absence of patient-level variables such as age, HIV status, nutritional status, and timing of treatment initiation the present analysis cannot disentangle these mechanisms or attribute mortality to specific causal pathways. This limitation underscores the importance of interpreting mortality patterns within a broader systems and contextual framework, rather than attributing observed differences to isolated clinical determinants. The findings are consistent with a growing body of evidence demonstrating that rural health systems face disproportionate challenges in both the delivery and monitoring of DR-TB care [2,12,13,29,32]. Structural constraints including workforce shortages, long travel distances, fragmented referral pathways, and limited access to specialist oversight reduce health systems’ capacity to ensure continuity of care, effective patient tracking, and timely outcome assignment [12,14]. These constraints are particularly pronounced in decentralized models of care, where responsibilities for diagnosis, treatment, and reporting are distributed across multiple levels of the health system.

In such settings, accountability for treatment outcome reporting is typically assigned to the final treating facility, often a resource-constrained primary or district-level service. This arrangement can create bottlenecks in outcome ascertainment, particularly where inter-facility coordination and data integration mechanisms are weak [29,33]. As a result, patients who transfer between facilities, disengage from care, or experience undocumented outcomes may not be systematically captured within routine surveillance systems. Within this context, the accumulation of outcomes classified as “not evaluated” is more plausibly interpreted as a manifestation of system-level capacity limitations rather than individual-level non-compliance or neglect. These findings highlight persistent structural inequities in program accountability between urban and rural settings, where differences in infrastructure, workforce capacity, and information systems contribute to uneven surveillance performance. Importantly, these patterns reinforce the need to interpret DR-TB treatment outcomes within their operational and health-system contexts. Assumptions of uniform surveillance quality across settings may lead to misinterpretation of program performance and obscure underlying governance gaps. A systems-based perspective integrating clinical, structural, and data-related dimensions is therefore essential for accurately assessing DR-TB outcomes and identifying priority areas for intervention.

Several alternative explanations may account for increasing proportions of “not evaluated” outcomes. These include delays in outcome assignment, incomplete data entry, patient movement between facilities, undocumented transfers, deaths occurring outside the formal healthcare system, and limitations in routine reporting processes. Because the available dataset did not contain information on these factors, the relative contribution of each explanation could not be assessed. Consequently, the observed patterns should not be attributed exclusively to governance or accountability challenges.

### 4.3. Implications for Performance Monitoring

MDR-TB cases exhibited higher observed rates of treatment failure and loss to follow-up (LTFU) compared with RR-TB cases. Although these differences did not reach statistical significance, they remain programmatically relevant. In the context of routine surveillance data, lack of statistical significance should not be interpreted as evidence of equivalence, but rather as a reflection of limited statistical power, small sample sizes, and variability in outcome reporting. Such patterns are consistent with existing evidence linking MDR-TB outcomes to greater treatment complexity, higher incidence of adverse drug effects, socioeconomic barriers to care, and weaknesses in patient follow-up systems within decentralized DR-TB programs [14,27,34]. Routine DR-TB outcome data form the basis of both domestic performance management systems and global accountability frameworks, including those coordinated by the World Health Organization. As such, incomplete outcome ascertainment has implications that extend beyond facility- or district-level evaluation, introducing uncertainty into national reporting and international benchmarking processes. TB control frameworks rely on accurate, complete, and timely indicators to assess progress, allocate resources, and evaluate policy effectiveness. Increasing proportions of outcomes classified as “not evaluated” therefore compromise the validity and comparability of key performance metrics, weakening the evidentiary foundation upon which accountability mechanisms are built [4,35,36]. Importantly, the presence of incomplete outcome data should not be viewed solely as a statistical limitation but as an indicator of underlying governance challenges within the health system. From a systems perspective, rising levels of “not evaluated” outcomes may signal deficiencies in patient tracking, data integration, reporting accountability, and inter-facility coordination [37,38,39]. These governance gaps can lead to systematic bias in reported outcomes, potentially masking true program performance and obscuring priority areas for intervention [37,39]. Accordingly, strengthening performance monitoring in DR-TB programs requires not only improved clinical care but also targeted investments in surveillance systems, data quality assurance, and accountability structures [37,38,40]. Interventions aimed at enhancing outcome completeness such as improved patient tracing mechanisms, integration of electronic health records, and clearer assignment of reporting responsibilities are essential to ensure that routine data accurately reflect program performance and support effective decision-making [39,40,41,42].

The operational consequences of incomplete outcome ascertainment extend beyond statistical uncertainty. For example, when patients are classified as “not evaluated,” programme managers cannot determine whether these individuals successfully completed treatment, died, transferred to another facility, or were lost to follow-up. This uncertainty can lead to inaccurate estimates of treatment success, distort facility performance indicators, and compromise comparisons across facilities and districts [38,39]. In practical terms, a facility reporting high treatment success with few documented failures may appear to perform well, while a substantial proportion of unevaluated cases could conceal adverse outcomes requiring intervention. Similarly, incomplete outcome reporting limits the ability of healthcare workers and programme managers to identify patients requiring tracing, assess continuity of care, and allocate resources effectively [41,42]. At the surveillance level, increasing numbers of unevaluated outcomes reduce confidence in routine programme indicators and may undermine evidence-based planning, monitoring, and accountability processes [37,39].

### 4.4. “Not Evaluated” as an Indicator of System Stress

Rather than treating “not evaluated” outcomes as a residual or non-informative category, the present findings support their interpretation as a sentinel indicator of system stress across patient tracking, follow-up, and data management processes. Increasing levels of missing outcome data may reflect cumulative strain within health systems, although alternative explanations such as delayed reporting, patient mobility, undocumented transfers, or data capture challenges cannot be excluded. From a systems perspective, these patterns may be viewed as potential early warning signals of declining surveillance functionality and weakened accountability structures. This reframing enhances the analytical utility of routine surveillance data. It aligns with implementation research demonstrating that adaptive responses in under-resourced health systems often manifest through shifts in data completeness and reporting patterns [14,27]. Moving beyond retrospective outcome classification toward forward-looking system diagnostics enables more responsive, adaptive program management. This approach is consistent with global health system performance frameworks that emphasize the use of tracer and early warning indicators to detect emerging failures in service delivery, continuity of care, and information systems before they translate into adverse clinical outcomes [2,38].

From an operational perspective, outcomes classified as “not evaluated” create uncertainty regarding the true status of patients within the programme. These patients may have transferred care, discontinued treatment, died, or completed treatment without appropriate documentation. Consequently, health systems lose the ability to accurately quantify treatment success, identify service gaps, and evaluate programme effectiveness. In resource-constrained settings, such information gaps may delay corrective actions, obscure emerging problems in patient retention, and weaken accountability for treatment outcomes.

### 4.5. Contribution to DR-TB and Health Systems Research

This study contributes to the DR-TB literature by extending analysis beyond patient-level determinants toward a systems-oriented interpretation of routine surveillance data. While existing research has predominantly focused on predictors of individual treatment outcomes, fewer studies have examined how surveillance governance, data completeness, and information system performance shape observed program trends, particularly in rural African contexts [27,33]. By conceptualizing DR-TB outcomes as governance signals, this study aligns with emerging calls to integrate health information systems, accountability mechanisms, and service delivery processes into program evaluation, particularly in resource-constrained rural settings such as the Eastern Cape [12]. It further positions routine DR-TB indicators as governance-sensitive measures that reflect both clinical care processes and the strength of underlying surveillance systems. This perspective is particularly relevant in limited-resource settings, where strengthening governance and data systems may yield substantial improvements in program performance alongside clinical interventions.

### 4.6. Policy Implications

The findings suggest that apparent deterioration in DR-TB outcomes may be influenced by incomplete outcome reporting and potential surveillance-related challenges, in addition to clinical factors. Fragmentation between key data systems, such as EDRWeb and the District Health Information System (DHIS), disrupts the continuity of patient records and delays or undermines outcome reporting, particularly in decentralized and rural settings. Emerging evidence from South Africa and globally suggests that integrated, case-based digital TB surveillance systems improve data completeness, strengthen accountability, and enhance the interpretability of program indicators. In contrast, reliance on parallel or aggregated systems obscures patient trajectories and distorts performance trends [30,39]. The rise in “not evaluated” outcomes also reflects deficiencies in patient tracing and community–facility linkage mechanisms. Evidence from rural South Africa highlights the critical role of community health workers in supporting patient retention, follow-up, and outcome ascertainment. However, their effectiveness is constrained when documentation systems are fragmented or poorly integrated with facility-level records [41,42]. Recent WHO guidance emphasizes outcome completeness as a core surveillance performance indicator and recommends the use of real-time digital dashboards to monitor delays, missing outcomes, and facility-level variation [30]. Compared with static reporting systems, governance-oriented dashboards facilitate earlier identification of system stress and enable more timely, targeted program responses. In resource-constrained settings, where accountability gaps can rapidly undermine the credibility of routine indicators, strengthening surveillance integration and data systems is essential for both program performance and policy decision-making.

### 4.7. Interpreting Findings Through the Governance Framework

This study operationalizes the CE–CG framework by demonstrating how routine program indicators can be reinterpreted as actionable governance signals. Across the 2020–2024 period, a clear system-level pattern emerged in which increasing proportions of outcomes classified as “not evaluated” coincided with apparent declines in treatment success, particularly in RR-TB and MDR-TB. Within the CE–CG framework, these patterns are interpreted as indicators of underlying health system performance rather than purely clinical outcomes. The marked increase in ‘not evaluated’ outcomes may be interpreted as a signal of system stress and may reflect challenges in patient tracing, follow-up, and data integration processes, although these pathways were not directly assessed. Global evidence indicates that incomplete outcome ascertainment is a key marker of weak health information systems, poor patient tracking, and disrupted continuity of care, particularly in resource-limited settings [2]. These dynamics are further shaped by structural constraints typical of rural health systems, including workforce shortages, fragmented referral pathways, and data management challenges, collectively representing potential governance-related challenges modes that affect both care delivery and surveillance reliability. From both global and rural health system perspectives, incomplete outcome reporting is increasingly understood as a signal of health system performance rather than clinical ineffectiveness. The WHO Global Tuberculosis Report identifies “not evaluated” outcomes as indicators of surveillance system weakness, particularly in settings affected by system shocks, resource constraints, and post-pandemic recovery challenges [2]. Similarly, rural health system analyses highlight failures in follow-up, referral coordination, and data integration as central drivers of incomplete outcomes, with patient attrition and data loss converging at the facility and district levels [36]. Importantly, global perspectives tend to emphasize functional system gaps such as delayed reporting and tracing failures. At the same time, rural analyses situate these issues within broader structural and governance constraints, including chronic staffing shortages, fragmented service delivery, and under-resourced data systems [40,43]. The TB Barometer reinforces this distinction, demonstrating that in rural contexts, incomplete outcomes are embedded within persistent governance challenges rather than short-term operational disruptions [36,40]. Together, these perspectives highlight that “not evaluated” outcomes represent a convergence point of clinical, structural, and governance failures.

## 5. Limitations

Findings from this study should be interpreted as exploratory and hypothesis-generating. The use of aggregated routine programme data precluded patient-level analyses and limited the ability to evaluate the influence of important demographic and clinical characteristics such as age, sex, HIV status, treatment history, treatment regimen, comorbidities, and socioeconomic factors on DR-TB outcomes. Consequently, the study was unable to identify patient-level predictors of treatment success, mortality, loss to follow-up, or incomplete outcome ascertainment.

The reliance on aggregated facility-level data also introduces the potential for ecological bias, whereby associations observed at the group level may not reflect relationships that exist at the individual patient level. As a result, observed trends should not be interpreted as evidence of individual-level risk factors or mechanisms. Furthermore, because the study design was retrospective, observational, and based on aggregated programme data, causal relationships between treatment outcomes, surveillance completeness, and governance-related factors cannot be established.

The analysis was restricted to two facilities in the Eastern Cape, limiting generalizability, although this focus intentionally reflects under-resourced rural health-system contexts. Small sample sizes, particularly for pre-XDR-TB and XDR-TB subgroups, reduced statistical precision, contributed to wide confidence intervals, and substantially limited the interpretability of inferential statistical testing. Consequently, findings for these subgroups should be regarded as descriptive and hypothesis-generating.

The increasing proportion of outcomes classified as “not evaluated” introduces potential reporting bias and complicates interpretation of temporal trends. Although bounding analyses were undertaken to explore the impact of missing-not-at-random (MNAR) data, these methods rely on assumptions that cannot be verified using the available dataset. Additionally, routine surveillance systems are subject to misclassification, delayed reporting, incomplete documentation, and inconsistencies across data sources, all of which may have influenced observed patterns.

Finally, the interpretation of increasing “not evaluated” outcomes as potential governance signals is conceptual and informed by the Community Engaged Clinical Governance framework. While the observed patterns are consistent with surveillance and accountability challenges described in the literature, governance processes were not directly measured in this study. Future research incorporating patient-level data, longitudinal follow-up, and direct measures of governance and surveillance performance is needed to validate and extend these findings.

## 6. Conclusions

DR-TB treatment outcomes in the Eastern Cape reflect not only clinical performance but also the interaction between care delivery, patient support systems, and surveillance governance. The observed decline in reported treatment success between 2021 and 2024 occurred alongside increasing proportions of outcomes classified as ‘not evaluated’. This pattern suggests that incomplete outcome ascertainment may have influenced reported programme indicators. This rise in incomplete outcome reporting is consistent with potential weaknesses in patient follow-up, tracking, and data systems, although these mechanisms were not directly examined in the present study. For example, among MDR-TB patients in 2024, 30.8% of outcomes were classified as ‘not evaluated’, meaning that nearly one-third of patients could not be definitively assigned to treatment success, failure, death, or loss to follow-up categories, substantially limiting interpretation of programme performance. These findings support further exploration of a systems-based perspective in which incomplete outcome reporting may serve as a potential governance signal. However, this interpretation remains conceptual and hypothesis-generating because governance processes were not directly measured. Future studies incorporating patient-level data, surveillance indicators, governance metrics, and direct assessment of accountability mechanisms are required to validate this proposed interpretation. Ultimately, strengthening surveillance systems is not only a data-quality priority but also a central pillar of effective DR-TB program governance.

## Figures and Tables

**Table 1:** Aggregate DR-TB Treatment Outcomes by Resistance Category (2020–2024).

TB Type	Started (*n*)	Success *n* (%)	Failed *n* (%)	LTFU *n* (%)	Died *n* (%)	Not Evaluated *n* (%)
MDR	178	120 (67.4)	10 (5.6)	18 (10.1)	15 (8.4)	12 (6.7)
Pre-XDR	23	17 (73.9)	2 (8.7)	1 (4.3)	3 (13.0)	0 (0.0)
RR	191	130 (68.1)	0 (0.0)	21 (11.0)	31 (16.2)	9 (4.7)
XDR	10	6 (60.0)	1 (10.0)	0 (0.0)	0 (0.0)	3 (30.0)

**Table 2:** RR-TB Treatment Outcomes by Year (2020–2024).

Year	Started (*n*)	Success % (95% CI)	Failed %	LTFU %	Died %	Not Evaluated %
2020	31	71.0 (53.4–83.9)	0.0	3.2	25.8	0.0
2021	44	79.5 (65.5–88.8)	0.0	6.8	13.6	0.0
2022	49	61.2 (47.2–73.6)	0.0	18.4	20.4	0.0
2023	36	75.0 (58.9–86.2)	0.0	13.9	8.3	2.8
2024	31	51.6 (34.8–68.0)	0.0	9.7	12.9	25.8

**Table 3:** MDR-TB Treatment Outcomes by Year (2020–2024).

Year	Started (*n*)	Success % (95% CI)	Failed %	LTFU %	Died %	Not Evaluated %
2020	34	67.6 (50.8–80.9)	11.8	17.6	0.0	0.0
2021	47	80.9 (67.5–89.6)	6.4	4.3	8.5	0.0
2022	39	66.7 (51.0–79.4)	5.1	10.3	17.9	0.0
2023	32	71.9 (54.6–84.4)	3.1	6.2	6.2	12.5
2024	26	38.5 (22.4–57.5)	0.0	15.4	7.7	30.8

**Table 4:** Pre-XDR-TB Treatment Outcomes by Year (2020–2024).

Year	Started (*n*)	Success % (95% CI)	Failed %	LTFU %	Died %	Not Evaluated %
2020	3	66.7 (20.8–93.9)	0.0	0.0	33.3	0.0
2021	5	80.0 (37.6–96.4)	20.0	0.0	0.0	0.0
2022	9	77.8 (45.3–93.7)	11.1	11.1	0.0	0.0
2023	3	66.7 (20.8–93.9)	0.0	0.0	33.3	0.0
2024	3	66.7 (20.8–93.9)	0.0	0.0	33.3	0.0

**Table 5:** XDR-TB Treatment Outcomes by Year (2020–2024).

Year	Started (*n*)	Success % (95% CI)	Failed %	LTFU %	Died %	Not Evaluated %
2020	1	100.0 (20.7–100.0)	0.0	0.0	0.0	0.0
2021	3	100.0 (43.8–100.0)	0.0	0.0	0.0	0.0
2022	3	66.7 (20.8–93.9)	33.3	0.0	0.0	0.0
2023	1	0.0 (0.0–79.3)	0.0	0.0	0.0	100.0
2024	2	0.0 (0.0–65.8)	0.0	0.0	0.0	100.0

**Table 6:** Key outcome trends and surveillance completeness comparisons.

TB Type	Outcome	Comparison	*p*-Value
RR-TB	Success	79.5% (2021) vs. 51.6% (2024)	0.075
RR-TB	Not evaluated	0% (2020–2022) vs. 25.8% (2024)	<0.001
MDR-TB	Success	80.9% (2021) vs. 38.5% (2024)	0.007
MDR-TB	Not evaluated	0% (2020–2022) vs. 30.8% (2024)	<0.001
Pre-XDR-TB	Success	66.7–80.0% across years	0.982
XDR-TB	Not evaluated	0% (2020–2022) vs. 100% (2023–2024)	0.040

Abbreviations: RR-TB, rifampicin-resistant tuberculosis; MDR-TB, multidrug-resistant tuberculosis; pre-XDR-TB, pre-extensively drug-resistant tuberculosis; XDR-TB, extensively drug-resistant tuberculosis. Footnote: Treatment success comparisons and trends in outcomes classified as “not evaluated” are presented to illustrate temporal changes in programme performance and surveillance completeness. *p*-values for pre-XDR-TB and XDR-TB should be interpreted with caution because of the very small sample sizes and limited statistical power.

## Data Availability

The data from this study are available upon request from the corresponding author.

## Abbreviations

The following abbreviations are used in this manuscript:

DR-TB	Drug-resistant tuberculosis
RR-TB	Rifampicin-resistant tuberculosis
MDR-TB	Multidrug-resistant tuberculosis
pre-XDR-TB	Pre-extensively drug-resistant tuberculosis
XDR-TB	Extensively drug-resistant tuberculosis
TB	Tuberculosis
LTFU	Loss to follow-up
WHO	World Health Organization
CE–CG	Community Engaged Clinical Governance
EDRWeb	Electronic Drug-Resistant Tuberculosis Register
DHIS	District Health Information System
CI	Confidence interval
MNAR	Missing not at random

## References

[1] Cilloni L., Kranzer K., Stagg H.R., Arinaminpathy N. (2020). Trade-offs between cost and accuracy in active case finding for tuberculosis: A dynamic modelling analysis. PLoS Med..

[2] World Health Organization (2023). Third WHO Consultation on the Translation of Tuberculosis Research into Global Policy Guidelines: Meeting Report.

[3] World Health Organization (2025). Global TB Report.

[4] South African National AIDS Council (SANAC) (2023). National Strategic Plan for HIV, TB and STIs 2023–2028. SANAC Website. https://sanac.org.za/wp-content/uploads/2023/05/SANAC-NSP-2023-2028-Web-Version.pdf.

[5] Saranjav A., Parisi C., Zhou X., Dorjnamjil K., Samdan T., Erdenebaatar S., Chuluun A., Dalkh T., Ganbaatar G., Brooks M.B. (2022). Assessing the quality of tuberculosis care using routine surveillance data: A process evaluation employing the Zero TB Indicator Framework in Mongolia. BMJ Open.

[6] Aminu-Ibrahim A., Ogbete J.C., Ambali K.B. (2020). Infrastructure Driven Expansion of Diagnostic Access Across Underserved and Rural Healthcare Regions. Int. J. Multidiscip. Res. Growth Eval..

[7] Ngene N.C., Khaliq O.P., Moodley J. (2023). Inequality in health care services in urban and rural settings in South Africa. Afr. J. Reprod. Health.

[8] Varshney K., Anaele B., Molaei M., Frasso R., Maio V. (2021). Risk factors for poor outcomes among patients with extensively drug-resistant tuberculosis (XDR-TB): A scoping review. Infect. Drug Resist..

[9] Kaapu K.G., Rukasha I. (2025). Trends and treatment outcomes of drug-resistant tuberculosis in Limpopo Province, South Africa (2011–2019): A Retrospective Study. PLoS ONE.

[10] Gebremariam R.B., Wolde M., Beyene A. (2021). Determinants of adherence to anti-TB treatment and associated factors among adult TB patients in Gondar city administration, Northwest, Ethiopia: Based on health belief model perspective. J. Health Popul. Nutr..

[11] Cox H., Salaam-Dreyer Z., Goig G.A., Nicol M.P., Menardo F., Dippenaar A., Mohr-Holland E., Daniels J., Cudahy P.G., Borrell S. (2021). Potential contribution of HIV during first-line tuberculosis treatment to subsequent rifampicin-monoresistant tuberculosis and acquired tuberculosis drug resistance in South Africa: A retrospective molecular epidemiology study. Lancet Microbe.

[12] Hosu M.C., Tsuro U., Dlatu N., Faye L.M., Apalata T. (2025). Strengthening clinical governance and public health interventions to improve drug-resistant tuberculosis outcomes in rural South Africa. Healthcare.

[13] Naidoo K., Perumal R., Cox H., Mathema B., Loveday M., Ismail N., Omar S.V., Georghiou S.B., Daftary A., O’Donnell M. (2024). The epidemiology, transmission, diagnosis, and management of drug-resistant tuberculosis lessons from the South African experience. Lancet Infect. Dis..

[14] Kielmann K., Dickson-Hall L., Jassat W., Le Roux S., Moshabela M., Cox H., Grant A.D., Loveday M., Hill J., Nicol M.P. (2021). We had to manage what we had on hand, in whatever way we could: Adaptive responses in policy for decentralized drug-resistant tuberculosis care in South Africa. Health Policy Plan..

[15] Le Roux S.R., Jassat W., Dickson L., Mitrani L., Cox H., Mlisana K., Black J., Loveday M., Grant A.D., Moshabela M. (2022). The role of emergent champions in policy implementation for decentralised drug-resistant tuberculosis care in South Africa. BMJ Glob. Health.

[16] Faye L.M., Hosu M.C., Apalata T. (2024). Drug-resistant tuberculosis in rural Eastern Cape, South Africa: A study of patients’ characteristics in selected healthcare facilities. Int. J. Environ. Res. Public Health.

[17] Said H., Ratabane J., Erasmus L., Gardee Y., Omar S., Dreyer A., Ismail F., Bhyat Z., Lebaka T., van der Meulen M. (2021). Distribution and clonality of drug-resistant tuberculosis in South Africa. BMC Microbiol..

[18] Tesfaye L., Lemu Y.K., Tareke K.G., Chaka M., Feyisa G.T. (2020). Exploration of barriers and facilitators to household contact tracing of index tuberculosis cases in Anlemo district, Hadiya zone, Southern Ethiopia: A qualitative study. PLoS ONE.

[19] Glasauer S., Kröger S., Haas W., Perumal N. (2020). International tuberculosis contact-tracing notifications in Germany: Analysis of national data from 2010 to 2018 and implications for efficiency. BMC Infect. Dis..

[20] Sachdeva D.A. (2024). Untangling the complex web of cutaneous tuberculosis in India: Addressing delays in diagnosis and treatment through strategic innovation and integrated care. Himal. J. Tuberc. Respir. Dis..

[21] Mokhethi N.P. (2023). Exploring Barriers to TB Contact Tracing and Screening: Health Service Providers’ Perspective. Doctoral Dissertation.

[22] Opperman M., Du Preez I. (2022). Factors contributing to pulmonary TB treatment lost to follow-up in developing countries: An overview. Afr. J. Infect. Dis..

[23] Barteka G., Bwayo D., Matovu J.K., Wanume B., Alunyo J.P., Sseguya R., Masaba J.P., Obbo J.S. (2025). Treatment outcomes and predictors of success for multidrug-resistant tuberculosis (MDR-TB) in Ugandan regional referral hospitals. Sci. Rep..

[24] Osman M., Meehan S.A., von Delft A., Du Preez K., Dunbar R., Marx F.M., Boulle A., Welte A., Naidoo P., Hesseling A.C. (2021). Early mortality in tuberculosis patients initially lost to follow up following diagnosis in provincial hospitals and primary health care facilities in Western Cape, South Africa. PLoS ONE.

[25] Kwabla M.P., Amuasi J.H., Krause G., Dormechele W., Takramah W., Kye-Duodu G., Osei E., Der J., Baiden F., Adusi-Poku Y. (2025). Completeness of tuberculosis case notification in Ghana: Record linkage and capture-recapture analysis of three TB registries. BMC Infect. Dis..

[26] Sinulingga H.E., Sinaga B.Y., Siagian P., Ashar T. (2023). Profile and risk factors of pre-XDR-TB and XDR-TB patients in a national reference hospital for Sumatra region of Indonesia. Narra J..

[27] Jassat W., Moshabela M., Nicol M.P., Dickson L., Cox H., Mlisana K., Black J., Loveday M., Grant A.D., Kielmann K. (2025). Decentralising DR-TB care: The trade-off between quality of care and service coverage in the early phase of implementation. Public Health Action.

[28] Chen J., Qiu Y., Wu W., Pan Y., Yang R., Li L., Yang Y., Lu K., Xu L. (2024). Incomplete tuberculosis reporting and registration to the surveillance system in southwestern China of Yunnan Province: An inventory survey. BMC Public Health.

[29] Iruedo J.O., Pather M.K. (2023). Lived experiences of patients and families with decentralised drug-resistant tuberculosis care in the Eastern Cape, South Africa. Afr. J. Prim. Health Care Fam. Med..

[30] World Health Organization (2024). Consolidated Guidance on Tuberculosis Data Generation and Use. Module 1. Tuberculosis Surveillance.

[31] Mulholland G.E. (2022). Using Enriched Clinical Cohort Data to Inform Interventions for TB Control in the Lake Victoria Region of East Africa. Doctoral Dissertation.

[32] Nxumalo E.L., Sineke N., Dlatu N., Apalata T., Faye L.M. (2025). Treatment outcomes of tuberculosis in the Eastern Cape: Clinical and socio-demographic predictors from two rural clinics. Int. J. Environ. Res. Public Health.

[33] Jassat W. (2022). The Decentralised Drug-Resistant TB Programme in South Africa: From Policy to Implementation.

[34] Nhassengo P.P. (2024). The TB-Poverty Cycle: Dynamics, Determinants, and Consequences of Economic Hardship Faced by People with TB in Mozambique. Doctoral Dissertation.

[35] Evans D.K. (2021). Understanding the Effects of the Global Fund TB grants on Tuberculosis Control Within the Health System of Ghana. Doctoral Dissertation.

[36] Ndlovu N., Padarath A.E. (2024). District Health Barometer 2022–23.

[37] South African Government (2023). National Strategic Plan for TB, HIV, and STIs 2023–2028.

[38] World Health Organization (2022). Global Tuberculosis Report 2021: Supplementary Material.

[39] Murphy J.P., Kgowedi S., Coetzee L., Maluleke V., Letswalo D., Mongwenyana C., Subrayen P., Charalambous S., Mvusi L., Dlamini S. (2022). Assessment of facility-based tuberculosis data quality in an integrated HIV-TB database in three South African districts. PLoS Glob. Public Health.

[40] Stop TB Partnership (2022). Global Plan to End TB 2023–2030.

[41] Malaka D.S., Lowane M.P. (2026). Beyond the clinic: Community health workers’ perspectives on tracing HIV and TB patients in rural South Africa. Open AIDS J..

[42] Chetty-Makkan C.M., DeSanto D., Lessells R., Charalambous S., Velen K., Makgopa S., Gumede D., Fielding K., Grant A.D. (2021). Exploring the promise and reality of ward-based primary healthcare outreach teams conducting TB household contact tracing in three districts of South Africa. PLoS ONE.

[43] Ntuli T.T. (2022). Socio-Economic Risk Factors for Acquired Multidrug-Resistant Tuberculosis Among Tuberculosis Patients in South Africa, Tshwane Health District: A Retrospective Study. Master’s Dissertation.

